# Melatonin Enhances Phenolics Accumulation Partially via Ethylene Signaling and Resulted in High Antioxidant Capacity in Grape Berries

**DOI:** 10.3389/fpls.2017.01426

**Published:** 2017-08-18

**Authors:** Lili Xu, Qianyu Yue, Feng’e Bian, Hong Sun, Heng Zhai, Yuxin Yao

**Affiliations:** State Key Laboratory of Crop Biology, Key Laboratory of Biology and Genetic Improvement of Horticultural Crops (Huang-Huai Region), Ministry of Agriculture, College of Horticulture Science and Engineering, Shandong Agricultural University Tai’an, China

**Keywords:** melatonin, polyphenols, antioxidant capacity, RNA-Seq, ethylene signaling, grape berries

## Abstract

This study assessed the primary impacts of exogenous melatonin (MT) treatment on grape berry metabolism. Exogenous MT treatment increased the endogenous MT content and modified berry ripening. Transcriptomic analysis revealed that the processes of polyphenol metabolism, carbohydrate metabolism and ethylene biosynthesis and signaling were the three most significantly altered biological processes upon MT treatment. Further experiments verified that MT treatment increased the contents of total anthocyanins, phenols, flavonoids and proanthocyanidins in berries. Additionally, the contents of 18 of the 22 detected individual phenolic compounds were enhanced by MT treatment; particularly, the resveratrol content was largely increased concomitantly with the up-regulation of *STS* gene expression. Meanwhile, MT treatment enhanced the antioxidant capacity of berries. On the other hand, it was indicated that ethylene participated in the regulation of polyphenol metabolism and antioxidant capacity under MT treatment in grape berries. In summary, MT enhances the polyphenol content and antioxidant capacity of grape berries partially via ethylene signaling.

## Introduction

Grapes (*Vitis vinifera* L) are one of the world’s largest fruit crops, with an annual production of more than 60 million metric tons. Approximately 71% of world grape production is used for wine, 27% as fresh fruit, and 2% as dried fruit. High-quality fruits are in high demand by consumers who value the role of fruits in maintaining and improving human well-being. The benefits of a diet rich in fruits to human health is related to the presence of bioactive compounds with antioxidant properties.

Melatonin (*N*-acetyl-5-methoxytryptamine) (MT) is an indoleamine that is synthesized from L-tryptophan metabolism via serotonin, and is a proven broad-spectrum antioxidant (Galano et al., 2011). Grapes and other fruits, such as apples, tomatoes, and so on, contain natural MT (Arnao and Hernández-Ruiz, 2014). MT has been shown to occur in all grape berry tissues (skin, flesh, and seeds) as well as in wine (Murch et al., 2010; Vitalini et al., 2011). Its concentration depends on the grape cultivar and the phenological stage; the highest concentration of MT in the vineyard occurs at the early stage of veraison in wine grapes (Murch et al., 2010). MT has been reported to have many physiological functions in plants, and its most frequently studied function is to prevent oxidative damage caused by various abiotic stresses such as salt (Li et al., 2017), chilling (Bałabusta et al., 2016), waterlogging (Zheng et al., 2017) and toxic metal cadmium (Hasan et al., 2015). Credible evidence suggests that MT should be classified as a mitochondria-targeted antioxidant; it achieves its antioxidant capacity via direct detoxification of reactive oxygen and reactive nitrogen species and indirectly by stimulating antioxidant enzymes while suppressing the activity of pro-oxidant enzymes (Zhang and Zhang, 2014; Reiter et al., 2016). Additionally, some studies indicate the role of MT in plants as a growth regulator and/or biostimulator (Arnao and Hernández-Ruiz, 2015). MT can affect root development by regulating auxin synthesis, transport and signaling in *Arabidopsis* and tomato (Wang et al., 2016; Wen et al., 2016), promote seed germination by regulating the biosynthesis and catabolism of ABA and GA4 in cucumber (Zhang et al., 2014), promote tomato ripening (Sun et al., 2015), and increase the size and synchronicity of grape berries (Meng et al., 2015).

Grapes, including the skin, pulp and seeds, contain a large amount of diverse phenolic compounds (Yilmaz et al., 2015). Red grape polyphenols are mainly flavonoid (anthocyanins, flavonols and flavanols, proanthocyanidins) and non-flavonoid compounds (phenolic acids, such as hydroxycinnamic and dydroxybenzoic acids and stilbenes/resveratrol), all known for their important biological actions (Monagas et al., 2005). The composition and concentration of phenolics in grapes vary with cultivars, species, viticultural and environmental factors (Yilmaz et al., 2015). Polyphenols represent the paradigm of the health-promoting effects ascribed to grape products (Monagas et al., 2005). Many studies have shown that polyphenols as strong antioxidants play a key role in preventing oxidative damage; e.g., polyphenols exert a strong antioxidative damage as radical scavengers *in vitro* (Monagas et al., 2005). Additionally, increasing evidence shows that the *in vivo* antioxidant effects of polyphenols arise from their ability to modulate cellular signaling transduction (Zhang and Tsao, 2016). A high correlation between phenolic composition and the antioxidant capacity of wine has been described, and anthocyanins are the main family with significant contributions to antioxidant capacity (Lingua et al., 2016a). Additionally, polyphenols make a positive contribution to antioxidant capacity, probably by synergism between phenolic compounds (Lingua et al., 2016b). For example, resveratrol is widely known as an antioxidant and the combination of resveratrol and other wine polyphenols leads to optimum antioxidant activity (Cavallini et al., 2016).

To date, it is largely unknown which metabolisms are altered by MT in fruit and whether MT can modify the metabolism of phenolic compounds and thereby alter the antioxidant capacity of grapes, although it was reported that MT increases total anthocyanin production in cabbages and tomatoes (Sun et al., 2015; Zhang et al., 2016). In this study, the ‘Moldova’ grape, which is widely planted as a table grape cultivar due to its high resistance to downy mildew in China, was used to explore the key metabolic changes in response to MT treatment and the possible mechanisms involved. This research will promote the application of melatonin for quality improvements in grape berries and additionally favoring revealing the mechanism underlying the regulation of MT on fruit metabolism.

## Materials and Methods

### Plant Materials and Treatments

The present experiment was undertaken at an experimental vineyard in Tai-An City, Shandong Province, China. Five- and 6-year-old self-rooted ‘Moldova’ vines were used in 2015 and 2016, respectively. The vines were planted at a row × vine spacing of 2.5 m × 2.0 m. Each vine had 15 vertical fruiting shoots on the horizontal cordon and shoots were topped to 12 nodes. When the Soluble Solid (TSS) of the berry reached 9.0° Brix (onset of veraison), the berries were subjected to the first MT treatment in 2015. The grape clusters on the vine were soaked for 10 s in a solution of 100 μM MT plus 0.05% (v/v) Triton X-100, which promoted MT absorption as a non-ionic surfactant. Eight days later, the second MT treatment was performed using the same method. CK berries were soaked in 0.05% (v/v) Triton X-100. At 0, 3, 7, 14, 24, 33, 42, 55, and 63 days after the first MT treatment (DAT), the berries were collected, frozen in liquid nitrogen, and stored at -70°C for the assays of berry ripening, phenolic compounds, antioxidant capacity and/or RNA-Seq. In a different experiment conducted in 2016, the same MT treatments as in 2015 were applied to determine statistical effects of year, MT treatment and their interaction on polyphenol content and antioxidant capacity; additionally, 100 μM MT, 100 μM MT + 5 μl.l^-1^ 1-MCP + 0.1% NiCl_2_ (w/v), and 100 μM ACC were applied at onset of veraison, and the berries were collected at 3 and 60 DAT to determine the role of ethylene in mediating MT signaling. The experiment was a randomized block design with three replications. Each replication consisted of 12 vines. Each fruiting shoot was controlled to produce two clusters. For each cluster, 15 berries were randomly sampled from the shoulder, middle, and tail.

### TSS and Titratable Acid (TA) Assay

The berry pulp was mixed, ground to homogenate, and centrifuged at 12, 000 rpm for 15 min. The supernatant was used to determine TSS and TA. TSS was determined using a digital refractometer (TD-40, TOP Instrument, Zhejiang, China). TA was determined by the titration of 10 ml of fruit juice with 0.1 M NaOH to an end point at pH 8.3.

### Soluble Sugar and Organic Acid Determinations

Soluble sugar and organic acid were extracted and determined using a capillary electrophoresis system (Beckman P/ACE, Fullerton, CA, United States) according to our previous method (Li et al., 2013).

### Extraction of Total Anthocyanins, Phenols, Phenolic Compounds and MT

Total anthocyanin, phenols and phenolic compounds were extracted from grape (skin and pulp) according to a previously reported method (Xu et al., 2011) with some modifications. One gram of ground skin or pulp was mixed with 8 mL acidified methanol (0.1% HCl, v/v) and sonicated in an ultrasonic bath for 15 min. After the supernatant had been poured out, the precipitate was extracted with 8 ml of the same solvent two more times. The supernatants were combined in a 50 mL tube and centrifuged at 5, 000 rpm for 15 min. The supernatant was then filtered on a filter paper, and the filtrate was evaporated to dryness at 30°C in a rotary evaporator. The residue was dissolved in 5 mL chromatographic grade methanol. During the extraction, the tubes were covered to minimize light exposure. Extractions were performed in three replicates for each individual powder-solvent combination.

Melatonin was extracted as previously reported (Sun et al., 2015) with some modifications, and the process was similar to polyphenol extraction but with the following changes: extraction was carried out under dim green light, acidified methanol was replaced by methanol, and the final extracts were transferred to a C_18_ solid phase extraction (SPE) cartridge (ProElut^TM^, DIKMA, China) for the purification of melatonin.

### Photometric Determination of Total Phenols, Flavonoids, Proanthocyanidins, and Anthocyanins

Total polyphenols were spectrophotometrically measured using the Folin–Ciocalteu method (Dewanto et al., 2002) with gallic acid as the standard. Total flavonoid and anthocyanin content was determined using the colorimetric method as previously reported (Dewanto et al., 2002) with rutin as the standard. Total proanthocyanidin content was measured using the vanillin assay (Sun et al., 1998) with vanillin as the standard.

### Identification of Anthocyanins by UPLC-QToF-MS

Anthocyanins were identified by ultra-performance liquid chromatography (UHPLC) coupled to quadrupole-time-of-flight mass spectrometry (Q-ToF-MS) (Waters Corp., Milford, MA, United States). The parameters and conditions were set following a previous description (Machado et al., 2015) with some modifications. The chromatographic separation was performed on a reverse-phase C_18_ analytical column (Acquity UPLC BEH C_18_, Waters) of 100 mm × 2.1 mm and 1.7 μm particle size. The injection volume was set to 5 μL. The column temperature was 45°C. Solvent A was acetonitrile, and solvent B was formic acid/water (2:98). The solvent flow rate was 0.3 mL/min, and the gradient was as follows: 0–20 min, 6–16% A; 20–28 min, 16–23% A; 28–35 min, 23–50% A; 35–37 min, 50% A; 37–40 min, 50–60% A. Quantitative analysis of the individual anthocyanins was carried out using an electrospray source operating in positive ionization mode under the following conditions: desolvation gas flow, 700 L/h; desolvation temperature, 400°C; cone gas flow, 10 L/h; source temperature, 100°C; capillary voltage, 3.0 kV; cone voltage, 30 V; collision energy, 25.0 eV. The full-scan mode was used (m/z, 50–2000). The amount of anthocyanin was calculated with reference to the external calibration curve of malvidin-3-*O*-glucoside.

### Identification of Non-flavanoid Phenolic Compounds and Flavanols by HPLC

Determination was performed as previously reported (Katalinić et al., 2010) with some modifications. Phenolic compounds were analyzed on a HPLC system (Waters 600, Waters, Milford, MA, United States) equipped with a Waters 2487 dual aaa absorbance detector. The extracts were filtered through 0.22-μm syringe filters and directly injected through a 10 μL fixed loop into a C_18_ guard column. Phenolic compounds were separated on an XDB-C18 column (4.6 mm × 250 mm, 5 μm, Kromasil, Sweden) maintained at 30°C. A gradient consisting of solvent A (water/acetic acid, 98:2) and solvent B (acetonitrile) was applied at a flow rate of 1.0 mL/min as follows: 0 min, 90% A and 10% B; 30 min, 65% A and 35% B; 42 min, 90% A and 10% B; 45 min, 90% A and 10% B. The signal was monitored at 280 nm. The phenolic compounds were quantified from the areas of their peaks at 280 nm using external standard calibration curves.

### MT Determination Using UHPLC-MS

The samples were separated on an Acquity UHPLC system (Waters, Milford, MA, United States) equipped with autosampler injection and pump systems (Waters). The separation was performed by injecting 10 μL of sample onto an ACQUITY UHPLC BEH C_18_ column (Waters, 2.1 mm internal diameter × 50 mm length, and 1.7 μm particle size). Mass spectrometry analyses were performed using a QTof-Micro mass spectrometer (Waters, Milford, MA, United States). The parameters and conditions of UHPLC-MS analysis were set according to a previous report (Gomez et al., 2012) with the exception of the following changes. The mobile phases consisted of water with 0.05% (v/v) of acetic acid (A) and methanol (B) delivered at 0.2 mL/min. The elution started at a composition of 90% A and 10% B; a 5 min linear gradient to 45% A, held for 2 min; return to the initial ratio of A and B by a 4 min gradient.

### Assays of Antioxidant Properties

Free radical scavenging activity was assessed by the DPPH assay based on the method of Katalinić et al. (2010). Antiradical activity was defined as the amount of antioxidant necessary to decrease the initial DPPH concentration by 50% (EC_50_). EC_50_ was calculated with gallic acid as equivalent; for reasons of clarity, the results were provided as antiradical power (ARP = 1/EC50). For the free radical scavenging activity assessed by the ABTS assay, a previous reported procedure was used (Re et al., 1999). The determination of ferric reducing antioxidant power (FRAP assay) was performed according to the method of Sun et al. (2011). For ABTS and FRAP assays, the radical scavenging activities of the samples were expressed as Trolox equivalent antioxidant capacity.

### Determinations of Ethylene Production Rate and ACC Content

Ethylene production rate was measured with a GC-9A gas chromatograph (Shimadzu, Japan) equipped with a GDX-502 column and a flame ionization detector (FID). Each of 30 fresh berries was enclosed in a 500-mL jar and incubated for 3 h. A 5-mL sample of the head-space gas was assayed. ACC content was determined using a previous method (Lizada and Yang, 1979).

### RNA-Seq and Real-time Quantitative PCR

Total RNAs were extracted using TRIzol Reagent (Invitrogen, Carlsbad, CA, United States) and mRNAs were purified from total RNAs using poly-T oligo-attached magnetic beads. Sequencing libraries were generated using NEBNext^®^ Ultra^TM^ RNA Library Prep Kit for Illumina^®^(#7530L, NEB, United States) following the manufacturer’s recommendations and index codes were added to attribute sequences to each sample. RNA concentration of library was measured using the Qubit^®^3.0 Fluorometer (Life Technologies, Carlsbad, CA, United States), and insert size was assessed using the Agilent Bioanalyzer 2100 system (Agilent Technologies, Santa Clara, CA, United States). After RNA concentration of library and insert size were assessed, the clustering of the index-coded samples was performed on a cBot cluster generation system using HiSeq PE Cluster Kit v4-cBot-HS (Illumina) according to the manufacturer’s instructions. After cluster generation, the libraries were sequenced on an Illumina Hiseq 2500 platform and 50-bp reads were generated. Clean reads were assembled into transcripts using Cufflinks with the grape genome^1^ as a reference. Unigene expression levels were quantified using reads per fragments per kilobase of transcript per million mapped reads (RPKM). Unigenes differentially expressed between two samples were screened using false discovery rate <0.05 and absolute log_2_ (fold changes) ≥ 1 as the threshold. The three replicates were conducted for each sample. Real-time quantitative PCR was performed using SYBR Green MasterMix (SYBR Premix EX Taq TM, Dalian, China) on a BIO-RAD iQ5 (Hercules, CA, United States) instrument, and the primers were listed in Supplementary Table 1.

### Statistical Analysis

All statistical analysis was performed by SPSS (V19.0) software. A one-way analysis of variance (ANOVA) followed by Duncan’s multiple range test was employed, standard deviation (SD) was calculated from three replicates. The differences between individual means were deemed to be significant at *p* < 0.05. General linear model was employed to determine the statistical effects of design variables (year and MT) on the tested parameters.

## Results

### MT Treatment Affects Berry Ripening

**FIGURE 1:**
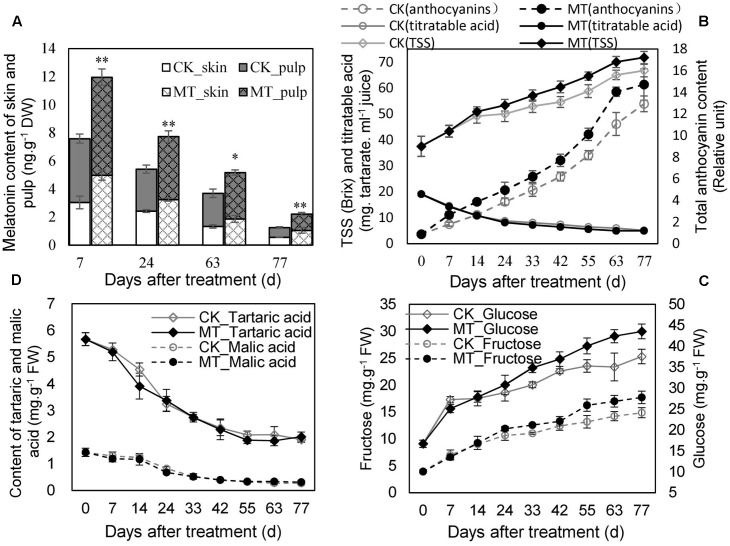
Changes in melatonin (MT) content **(A)** and the parameters related to berry ripening **(B–D)** in the CK and MT-treated berries during ripening. Grape berries were treated with 100 μM MT at veraison. Values represent the means ± SD of three replicates. ^∗^ Significant difference, *p* < 0.05; ^∗∗^ highly significant difference, *p* < 0.01.

The MT content in the berry (skin and pulp) was determined at 7, 24, 63, and 77 days after treatment (DAT). MT treatment significantly enhanced the endogenous MT content of the berries, although MT declined gradually along with ripening in the CK- and MT-treated berries (MT berries) (**Figure 1A**). The MT berries accumulated more total anthocyanins and sugars (TSS, glucose and fructose) than the CK berries at the same DAT from 24 DAT (**Figures 1B,C**), indicating that MT treatment promoted berry ripening. In contrast, titratable, tartaric, and malic acids were not clearly altered by MT treatment (**Figures 1B,D**). Therefore, MT selectively modified maturity-related parameters.

### The Clearly Altered Pathways in Grape Berries Induced by MT Treatment

**FIGURE 2:**
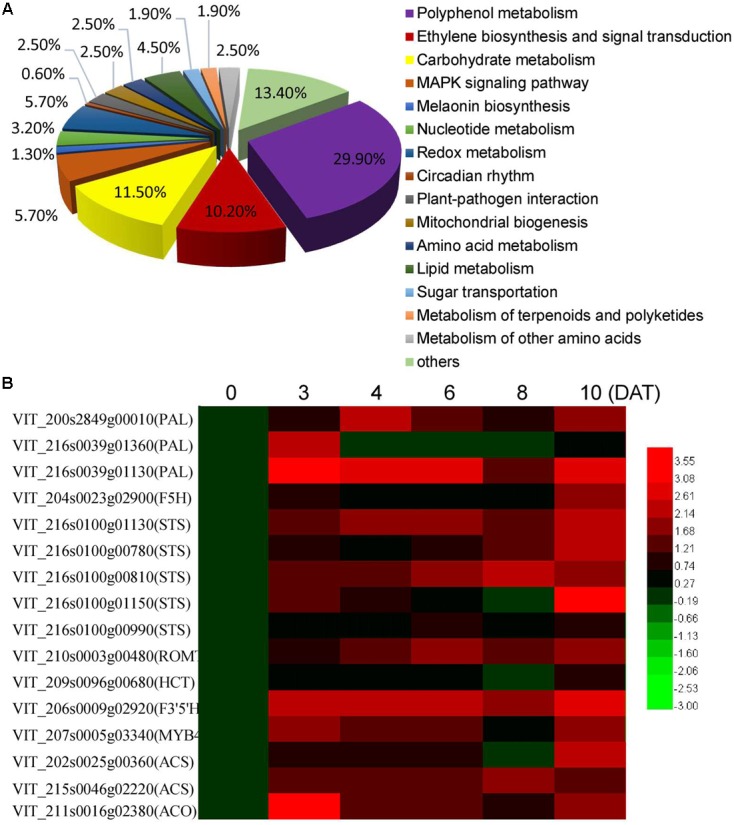
Biological process of the differentially expressed genes classified by gene ontology **(A)** and the expression levels of the genes related to polyphenol metabolism and ethylene biosynthesis at different days after MT treatment (DAT) **(B)**. In **(A)**, statistics of pathway enrichment of DEGs were conducted using RichFactor and *q*-value. RichFactor is the ratio of differentially expressed gene number to all gene number in this pathway term. Greater RichFator means greater intensiveness. *Q*-value is corrected *p*-value ranging from 0 to 1, and less *q*-value means greater intensiveness. The top 15 of enriched pathway terms were displayed in this paper. In the heat map shown in **(B)**, the qRT-PCR fold-change is calculated by comparing the relative expression values of the selected genes in the MT and CK berries, and log_2_ (fold change) is used to form the heat map. PAL, phenylalanine ammonia-lyase; STS, Stilbene synthase; ROMT, *Trans*-resveratrol di-*O*-methyltransferase; HCT, Shikimate *O*-hydroxycinnamoyltransferase; F3′5′H, flavonoid 3′,5′-hydroxylase; ACS, 1-aminocyclopropane-1-carboxylate synthase; ACO, 1-aminocyclopropane-1-carboxylate oxidase.

To further explore the key changes in berry metabolism induced by MT, RNA-Seq analysis of the CK and MT berries at 3 DAT was performed to quantify gene changes. It was found that 567 and 262 genes were up- and down-regulated by at least onefold in the MT berries, respectively (Supplementary Table 2). According to the putative homology to sequences present in public databases, all of the annotated DGE genes were associated with 15 biological processes. The processes of polyphenol metabolism, carbohydrate metabolism, ethylene biosynthesis and signaling, MARK signaling pathway, and redox metabolism were clearly changed (**Figure 2A** and Supplementary Table 2). The process of polyphenol metabolism contained the most DGE genes (29.9%), including 31 *stilbene synthases* (*STS*), 7 *phenylalanine ammonia-lyases* (*PALs*) and some other genes related to polyphenol metabolism, such as *MYB4*, *trans-resveratrol di-o-methyltransferase*, and *flavonoid 3,5-hydroxylase* (Supplementary Table 2). Therefore, it was suggested that polyphenol metabolism and especially resveratrol synthesis are strongly altered by MT. Additionally, 15 genes related to ethylene biosynthesis and signaling, including two *ACSs*, two *ACOs* and 11 *ethylene-responsive transcription factors (ERFs*), were upregulated by MT treatment (Supplementary Table 2), suggesting that MT modifies ethylene synthesis and signaling. Moreover, the expression levels of 16 genes related to polyphenol metabolism and ethylene biosynthesis were detected by qRT-PCR to validate the reliability of the DGE genes and further to elucidate the expression patterns at different time points after MT treatment (**Figure 2B**). All of the detected genes were up-regulated to varying extents at 3 DAT. Additionally, the expression of 12 genes was continuously induced by MT from 3 to 10 DAT, indicating the lasting influence of MT on polyphenol metabolism. It should be noted that the expression level was clearly enhanced at 10 DAT (2 days after the second MT application), compared with 8 DAT, suggesting the dose influence of MT on gene expression.

### MT Treatment Increases the Content of Total Phenols, Flavonoids, and Proanthocyanidins of Berries

Accumulations of total phenols, flavonoids and proanthocyanidins were determined to further evaluate the changes in polyphenol metabolism induced by MT. The contents of total phenols, flavonoids and proanthocyanidins were gradually enhanced along with berry ripening and reached the maximum levels at 77 DAT in berry skin (**Table 1**). In contrast, total phenols, proanthocyanidins and especially flavonoids were present at low concentrations, and total phenols and flavonoids remained stable with ripening in the pulp (**Table 1**). Compared to the CK berries, MT treatment generally enhanced the content of total phenols, flavonoids and proanthocyanidins at the three stages in a statistically significant manner. The maximum increments of 22.4, 14.2, and 21.4% were found for total phenols, flavonoids and proanthocyanidins, respectively, in the MT berries (sum of skin and pulp) compared with the CK berries at 63 DAT (**Table 1**).

**Table 1:** Contents of total phenols, flavonoids and proanthocyanidins in berry skin and pulp extracts.

		Total phenols (mg gallic acid g^-1^ FW)		Total flavonoids (mg rutin g^-1^ FW)		Total proanthocyanidins (mg vanillin g^-1^ FW)	
Sample	DAT	CK	MT	CK	MT	CK	MT
Pulp	42	0.18 ± 0.01	0.22 ± 0.01*	0.09 ± 0.01	0.10 ± 0.01	4.14 ± 0.70	7.44 ± 0.24**
	63	0.18 ± 0.02	0.23 ± 0.02*	0.09 ± 0.003	0.13 ± 0.03*	8.62 ± 0.27	11.06 ± 0.42**
	77	0.19 ± 0.01	0.27 ± 0.02**	0.10 ± 0.01	0.13 ± 0.01*	10.83 ± 0.84	13.48 ± 1.22**
Skin	42	3.31 ± 0.37	3.54 ± 0.40	12.88 ± 1.04	14.07 ± 1.39	70.41 ± 5.93	77.82 ± 3.12*
	63	3.65 ± 0.27	4.91 ± 0.61*	13.10 ± 1.05	14.93 ± 1.54*	80.32 ± 1.62	96.95 ± 6.99**
	77	4.22 ± 0.26	5.31 ± 0.56*	17.90 ± 1.71	19.18 ± 1.23*	84.62 ± 2.39	113.66 ± 8.56**

*Values are reported as the means ± SD. DAT, days after treatment. ^∗^Significant difference, *p* < 0.05; ^∗∗^ highly significant difference, *p* < 0.01*.

### MT Treatment Modifies Phenolic Compound Profiles of Berries

To investigate the changes in the phenolic compound contents between the CK and MT berries, 22 phenolic compounds were determined (**Tables 2**, **3**). A total of nine non-flavonoid phenolic compounds were detected in berry pulp and skin, of which only phloretin declined with berry ripening. The skin accumulated much higher concentrations of non-flavonoid phenolic compounds than the pulp (**Tables 2**, **3**). Coumaric acid was the most abundant phenolic compound, followed by gallic acid, ferulic acid and resveratrol, in grape skin at the three stages (**Table 3**). In contrast, chlorogenic acid exhibited the highest concentration in pulp, followed by gallic and caffeic acids, and the others were present at relatively low concentrations (**Table 2**). MT treatment significantly enhanced the contents of the detected non-flavonoid compounds with the exception of ferulic acid and phloretin, and produced more than 75.7 and 44.2% increments in the total non-flavonoid phenolic compounds in pulp and skin, respectively, at the three stages. In particular, the content of resveratrol was increased 3.56- and 0.98-fold by MT treatment in pulp and skin, respectively, at 63 DAT, corresponding to the expression up-regulation of large amounts of *STS* (Supplementary Table 2); additionally, caffeic, chlorogenic, and gallic acids were also largely enhanced by MT treatment (**Tables 2**, **3**).

**Table 2:** Content of non-flavonoid phenolic compounds and flavanols in the CK and MT berry pulps.

	DAT 42		DAT 63		DAT 77	
	CK	MT	CK	MT	CK	MT
**Non-flavonoid phenolic compounds (mg g^-1^ DW)**						
Chlorogenic acid	0.48 ± 0.02	0.80 ± 0.05^∗∗^	0.77 ± 0.05	2.14 ± 0.11^∗∗^	0.79 ± 0.01	2.49 ± 0.31^∗∗^
Gallic acid	0.07 ± 0.01	0.14 ± 0.02^∗∗^	0.56 ± 0.07	1.05 ± 0.15^∗∗^	0.55 ± 0.07	1.20 ± 0.18^∗∗^
Caffeic acid	0.06 ± 0.007	0.13 ± 0.02^∗∗^	0.13 ± 0.01	0.52 ± 0.012^∗∗^	0.36 ± 0.01	1.29 ± 0.02^∗∗^
Syringic acid	0.04 ± 0.006	0.08 ± 0.01^∗∗^	0.04 ± 0.003	0.07 ± 0.02^∗^	0.05 ± 0.006	0.06 ± 0.01
Coumaric acid	0.01 ± 0.002	0.03 ± 0.002^∗∗^	0.02 ± 0.003	0.04 ± 0.005^∗∗^	0.04 ± 0.003	0.07 ± 0.02^∗^
Feralic acid	0.02 ± 0.003	0.02 ± 0.004	0.04 ± 0.003	0.04 ± 0.006	0.02 ± 0.003	0.02 ± 0.002
Phloretin	0.05 ± 0.01	0.07 ± 0.01	0.04 ± 0.006	0.05 ± 0.009	0.04 ± 0.006	0.05 ± 0.007
Cinnamic acid	0.08 ± 0.005	0.11 ± 0.002^∗^	0.23 ± 0.004	0.33 ± 0.003^∗∗^	0.09 ± 0.01	0.13 ± 0.002^∗∗^
*Trans*-resveratrol	0.04 ± 0.003	0.08 ± 0.006^∗∗^	0.07 ± 0.008	0.31 ± 0.004^∗∗^	0.10 ± 0.02	0.46 ± 0.01^∗∗^
SUM	0.84	1.48	1.90	4.87	2.03	5.75
**Flavanols (mg g^-1^ DW)**						
(-)Epicatechin	8.92 ± 1.19	12.52 ± 1.17^∗^	7.79 ± 0.73	9.85 ± 0.87^∗^	3.86 ± 0.22	7.42 ± 0.33^∗∗^
(+)Catechin	0.53 ± 0.06	0.99 ± 0.13^∗∗^	1.36 ± 0.02	2.01 ± 0.03^∗∗^	1.58 ± 0.02	2.46 ± 0.08^∗∗^
SUM	9.45	13.51	9.15	11.86	5.44	9.88

*Values are reported as the means ± SD. DAT, days after treatment. ^∗^Significant difference, *p* < 0.05; ^∗∗^ highly significant difference, *p* < 0.01*.

**Table 3:** Content of non-flavonoid phenolic compounds, flavanols and anthocyanin compounds in the CK and MT berry skins (Unit: mg g^-1^ DW).

	DAT 42		DAT 63		DAT 77	
	CK	MT	CK	MT	CK	MT
**Non-flavonoid phenolic compounds**						
Chlorogenic	0.33 ± 0.04	0.62 ± 0.05^∗∗^	0.70 ± 0.02	1.24 ± 0.19^∗∗^	0.90 ± 0.05	1.19 ± 0.23^∗^
Gallic acid	0.88 ± 0.09	1.52 ± 0.21^∗∗^	1.44 ± 0.16	2.65 ± 0.22^∗∗^	1.50 ± 0.13	2.85 ± 0.24^∗∗^
Caffeic acid	0.38 ± 0.05	0.99 ± 0.14^∗∗^	0.78 ± 0.10	1.36 ± 0.30^∗^	1.90 ± 0.18	2.83 ± 0.24^∗∗^
Syringic acid	0.12 ± 0.25	0.31 ± 0.05^∗^	0.37 ± 0.05	0.43 ± 0.05	0.36 ± 0.04	0.31 ± 0.05
Coumaric acid	3.40 ± 0.85	5.07 ± 0.82^∗^	4.82 ± 0.61	6.25 ± 0.67^∗∗^	5.83 ± 0.48	7.72 ± 0.90^∗∗^
Feralic acid	0.46 ± 0.11	0.53 ± 0.61	1.23 ± 0.17	1.23 ± 0.09	0.71 ± 0.05	0.71 ± 0.08
Phloretin	1.27 ± 0.23	1.65 ± 0.32	1.00 ± 0.12	1.11 ± 0.25	0.49 ± 0.02	0.58 ± 0.11
Cinnamic acid	1.15 ± 0.21	1.91 ± 0.25^∗^	1.00 ± 0.20	1.54 ± 0.10^∗^	1.51 ± 0.11	2.20 ± 0.17^∗∗^
*Trans*-Resveratr	1.47 ± 0.12	2.79 ± 0.33^∗∗^	1.15 ± 0.16	2.28 ± 0.31^∗∗^	1.04 ± 0.12	2.02 ± 0.15^∗∗^
SUM	9.46	15.43	12.46	18.10	14.24	20.44
**Flavanols**						
(-)Epicatechin	8.47 ± 1.25	10.59 ± 1.65^∗^	3.78 ± 0.27	7.21 ± 0.44^∗∗^	1.62 ± 0.14	2.67 ± 0.19^∗∗^
(+)Catechin	1.05 ± 0.06	1.78 ± 0.21^∗∗^	1.82 ± 0.20	2.64 ± 0.13^∗^	3.94 ± 0.22	4.17 ± 0.43
SUM	9.52	12.37	5.61	9.84	5.57	6.84
**Anthocyanin compounds**						
Mv-3,5-Glu	7.25 ± 0.85	22.65 ± 1.35^∗∗^	15.20 ± 1.10	27.95 ± 3.95^∗∗^	27.50 ± 2.51	29.25 ± 3.25
Mv-3-Glu	1.21 ± 0.15	2.12 ± 0.25^∗∗^	1.83 ± 0.14	2.25 ± 0.12^∗^	2.84 ± 0.31	3.43 ± 0.24^∗^
Pn-(6-Caff)Glu	0.74 ± 0.11	2.16 ± 0.27^∗∗^	1.71 ± 0.13	6.25 ± 0.86^∗∗^	4.05 ± 0.24	3.17 ± 0.44^∗^
Pn-(3-AC)Glu	0.13 ± 0.01	0.15 ± 0.01	0.05 ± 0.002	0.07 ± 0.01	0.05 ± 0.001	0.06 ± 0.01
Pn-3-Glu	0.15 ± 0.01	0.15 ± 0.01	0.15 ± 0.01	0.19 ± 0.02	0.31 ± 0.02	0.36 ± 0.03
Pt-3,5-Glu	0.62 ± 0.05	2.06 ± 0.22^∗∗^	1.67 ± 0.22	5.02 ± 0.74^∗∗^	2.48 ± 0.15	2.51 ± 0.23
Pt-(6-Coum)Glu	0.99 ± 0.05	1.94 ± 0.13^∗∗^	1.55 ± 0.27	2.38 ± 0.36^∗∗^	1.71 ± 0.14	2.98 ± 0.04^∗∗^
Pt-3-Glu	0.37 ± 0.04	0.61 ± 0.01^∗^	0.50 ± 0.03	0.98 ± 0.13^∗∗^	0.75 ± 0.11	1.46 ± 0.20^∗∗^
Dp-3-(6-Coum)	0.51 ± 0.07	1.08 ± 0.12^∗∗^	0.88 ± 0.06	1.43 ± 0.17^∗∗^	1.13 ± 0.16	2.48 ± 0.17^∗∗^
Dp-3-Glu	0.27 ± 0.02	0.43 ± 0.05^∗^	0.37 ± 0.03	0.50 ± 0.02^∗^	0.66 ± 0.02	1.23 ± 0.17^∗∗^
Cy-3-Glu	0.06 ± 0.004	0.05 ± 0.002	0.05 ± 0.005	0.12 ± 0.01^∗∗^	0.11 ± 0.01	0.17 ± 0.02^∗^
SUM	12.30	33.40	23.96	47.14	41.59	47.10

*Values are reported as the means ± SD. Mv, malvidin; Glu, glucoside; Coum, coumaroyl; Pn, peonidin; Caff, caffeoyl; AC, acetyl; Pt, Petunidin; Dp, delphinidin; Cy, cyanidin; DAT, days after treatment. ^∗^Significant difference, *p* < 0.05; ^∗∗^highly significant difference, *p* < 0.01*.

Two flavanols, catechin and epicatechin, were determined. Epicatechin gradually increased with berry ripening while catechin showed opposite changes in the pulp and skin. Catechin and epicatechin were present at a high concentration compared to non-flavonoid phenolic compounds in the pulp and skin (**Tables 2**, **3**). Generally, MT significantly elevated the content of catechin and epicatechin in the pulp and skin, and led to more than 30.0% increases in berries (skin and pulp) (**Tables 2**, **3**).

The anthocyanin profiles of the detected samples primarily consisted of malvidin (Mv), peonidin (Pn), petunidin (Pt), delphinidin (Dp), and cyanidin (Cy) (**Table 3**). Mv-3,5-glucose (Glu) was the most abundant anthocyanin and accounted for more than 59.2% of the detected total anthocyanins at different developmental stages. In contrast, Pn-(3-AC)Glu, Pn-3-Glu and Cy-3-Glu were present at relatively very low levels (**Table 3**). Most of the detected anthocyanins increased with berry ripeness and led to the large increase in total concentration in CK berries. In contrast, the MT berries accumulated the same levels of total content of anthocyanins at 63 and 77 DAV (**Table 3**). When comparing anthocyanin content between the CK and MT berries, all of the detected anthocyanins with the exception of Pn-(3-AC)Glu and Pn-3-Glu were enhanced by MT treatment, and the total content was increased 76.9% by MT treatment at 63 DAV. In contrast, only seven anthocyanins were significantly enhanced by MT, and only a 12.0% increase in total concentration was produced by MT treatment at 77 DAV (**Table 3**).

### MT Treatment Enhances the Antioxidant Capacity of Berries

The *in vitro* antioxidant activities of the CK and MT berries were evaluated by DPPH, ABTS, and FRAP assays. It was found that the skin had a much higher antioxidant capacity than pulp (**Table 4**), in accordance with high levels of phenolic compounds in skin (**Tables 2**, **3**). When comparing the antioxidant capacity between CK and MT berries, the MT berries had a higher antioxidant capacity than the CK berries at the three stages and reached significant levels in most samples (**Table 4**).

**Table 4:** Antioxidant activities of the CK and MT pulp and skin.

		DPPH (1/EC50) (unit of EC50: mg gallic acid equivalent g^-1^ FW)		ABTS (mg Trolox equivalent g^-1^ (FW)		FRAP (mg Trolox equivalent g^-1^ FW)	
Sample	DAT	CK	MT	CK	MT	CK	MT
Pulp	42	0.020 ± 0.003	0.025 ± 0.003	0.051 ± 0.002	0.055 ± 0.002	0.063 ± 0.007	0.073 ± 0.007*
	63	0.023 ± 0.001	0.037 ± 0.005**	0.045 ± 0.003	0.064 ± 0.006**	0.065 ± 0.007	0.094 ± 0.008**
	77	0.022 ± 0.002	0.032 ± 0.002**	0.053 ± 0.003	0.066 ± 0.005**	0.079 ± 0.004	0.093 ± 0.010*
Skin	42	0.17 ± 0.02	0.22 ± 0.03	64.45 ± 5.85	67.54 ± 1.45*	62.07 ± 5.6	66.74 ± 4.78*
	63	0.18 ± 0.02	0.25 ± 0.02*	64.36 ± 5.17	75.31 ± 7.22*	83.06 ± 7.07	102.85 ± 8.57**
	77	0.20 ± 0.01	0.27 ± 0.02*	66.64 ± 1.97	73.27 ± 1.79*	73.81 ± 2.49	106.41 ± 11.32**

*Values are reported as the means ± SD. DAT, days after treatment. ^∗^Significant difference, *p* < 0.05; ^∗∗^highly significant difference, *p* < 0.01*.

### Statistical Effects of Year, MT Treatment and Their Interaction on Polyphenol Content and Antioxidant Capacity

To investigate whether MT can impart repeatable effects on polyphenol content and antioxidant capacity of the berries, the polyphenol content, antioxidant activities and some parameters related to berry ripening were determined and evaluated in the berries at 63 DAT in 2015 and 2016 (Supplementary Table 3). The year affected 41.2% of the tested parameters in a statistically significant manner. In contrast, all of the parameters were significantly affected by the MT treatment. Additionally, the MT treatment produced even smaller *p*-values than year with regard to all of the parameters. Therefore, MT played a predominant role in determining the modifications of phenolic compounds and antioxidant capacity between the CK and MT berries, although some parameters were influenced by year.

### MT Enhances Polyphenol Accumulation and Antioxidant Activities of Berry Skins Partially via Increasing Ethylene Production

**FIGURE 3:**
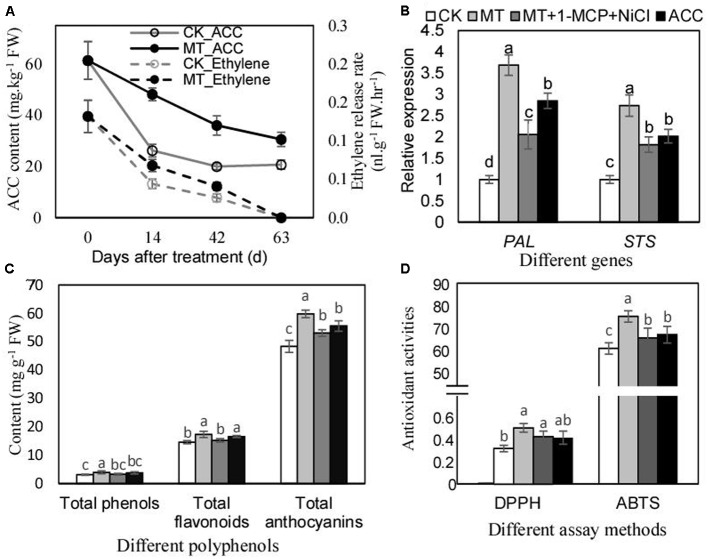
Ethylene production levels **(A)**, relative expression of *PAL* and *STS* **(B)**, total phenol content **(C)** and antioxidant activities of berry skins after MT and other treatments. The expression levels **(B)** were determined using the berry skins at 3 days after treatment; the contents of total phenols, flavonoids and anthocyanins **(C)**, and the antioxidant activities **(D)** were detected using the skins at 60 days after treatment. In **(B)**, the accession numbers of *PAL* and *STS* are VIT_216s0039g01130 and VIT_216s0100g00810, respectively. In **(C)**, the contents of total phenols, flavonoids, and anthocyanins were calculated using gallic acid, rutin and vanillin equivalents, respectively. In **(D)**, the units of DPPH and ABTS were 1/EC50 and mg Trolox equivalent g^-1^ FW, respectively. Values represent the mean of three replicates ± SD. The difference was not significant at 5% significance level among the values labeled with the same letter.

Ethylene biosynthesis and signaling were suggested to be promoted by MT (**Figures 2A,B**). The further studies were performed to investigate the role of ethylene in mediating the regulation of polyphenol mentalism under MT treatment. ACC content and ethylene production rate were remarkably increased by MT treatment, and the maximum increments of 83.7 and 54.5% were generated for ACC content and ethylene production rate, respectively, by MT treatment at 14 DAT (**Figure 3A**). Additionally, ACC treatment up-regulated the expression of *PAL* and *STS*, indicating the effects of ethylene on polyphenol metabolism. In contrast, MT had stronger effects on the expression of *PAL* and *STS*; however, MT treatment with a double block treatment of ethylene via NiCl_2_ plus 1-methylcyclopropene (1-MCP) reduced the effects of MT, indicating that MT regulated the expression of *PAL* and *STS* partially via ethylene (**Figure 3B**). Similar influences of ACC, MT, and MT+NiCl_2_+1-MCP treatments were found for the content of total phenols, flavonoids and proanthocyanidins (**Figure 3C**), as well as for antioxidant activities (**Figure 3D**). Taken together, MT treatment enhanced ethylene production, which partially contributed to polyphenol accumulations and antioxidant activities.

## Discussion

The role of MT in regulating berry ripening was indicated by the accelerating accumulation of sugars and anthocyanins (**Figures 1B,C**) as well as the differentially expressed genes related to carbohydrate metabolism (**Figure 2A** and Supplementary Table 2). Similar increases in TSS, glucose and pigment accumulations were also observed in MT-treated tomato fruits (Sun et al., 2015; Liu et al., 2016). However, it was found that titratable acidity and organic acids might be independent of MT treatment (**Figures 1B,D**). These observations suggest that MT likely acts as a ripening modulator rather than a trigger. Up to now, whether MT can modify fruit polyphenol metabolism remains largely unclear. Plant phenolics arise biogenetically from the shikimate/phenylpropanoids pathway; the key structure of the plant phenylpropanoids pathway consists of many regulatory and structure genes, such as *PAL*, *cinnamate 4-hydroxylase* (*C4H*), *stilbene synthase* (*STS*), *chalcone synthase* (*CHS*), and *UFGT*. The expression up-regulation of the genes related to the phenylpropanoid pathway suggested the regulation of MT on polyphenol metabolism in the MT-treated berries (Supplementary Table 2), cabbage seedlings (Zhang et al., 2016) and tomato fruits (Sun et al., 2015). Additionally, the altered phenolic compound profiles verified the MT-induced metabolism modifications of polyphenols (**Tables 2**, **3**). Moreover, in addition to the genes involved in polyphenol metabolism, the expression of large amounts of other genes, involved in ethylene biosynthesis, MAPK signaling, nucleotide metabolism and so on, was greatly modified by MT treatment (**Table 2** and Supplementary Table 2), suggesting that MT, probably as a signal molecule itself (Lee et al., 2014), induces massive transcriptional reprogramming.

The mechanisms involved in perceiving and signaling MT remain poorly understood. The published data indicates that MT can affect the levels of ABA and GA in seeds or leaves (Zhang et al., 2014). In contrast, we did not identify significantly changed genes involved in ABA metabolism and signaling in MT berries (Supplementary Table 2), although grape berries are classified as non-climacteric fruits, and ABA has been suggested to play an important role in modulating grape berry ripening (Sun et al., 2010). Instead, ethylene biosynthesis and signaling was the third largest biological process changed by MT (**Figure 1B**). Additionally, a few studies have shown that grape berry tissues have a fully functional pathway for ethylene synthesis and that this pathway is activated just before veraison, and ethylene perception is critical for some grape berry ripening (Sun et al., 2010). Moreover, MT treatment enhanced ethylene production via increasing ACC levels (**Figure 2A**). Therefore, ethylene participates in MT signaling and probably plays a key role in mediating MT-regulating quality modifications in non-climacteric grape fruits.

In addition, ethylene was reported to play a role in regulating polyphenol metabolism (Becatti et al., 2014). In this study, ACC treatment did promote the expression of *PAL* and *STS* and the accumulation of polyphenols (**Figures 3B,C**). Similarly, higher concentrations of specific phenolic compounds belonging to the classes of flavonols, anthocyanins, flavan-3-ols, and stilbenes are detected in the wine made from postharvest ethylene-treated berries; additionally, postharvest ethylene treatment activates the expression of the genes related to the phenylpropanoid pathway in grape flesh (Becatti et al., 2014). Additionally, MT treatment with a double block treatment of ethylene via NiCl_2_ plus 1-MCP reduced the effects of MT on polyphenol content (**Figure 3C**). Taken together, ethylene participated in the MT-induced regulation of polyphenol metabolism. However, the ethylene-mediated pathway only partially explained the role of MT in enhancing polyphenol accumulation (**Figures 3B–D**); unknown pathways via which MT regulates polyphenol metabolism are supposed to exist.

In this study, MT treatment conferred grape berry with high free radical scavenging capacity and reducing antioxidant power indicated by DPPH, ABTS, and FRAP assays (**Table 4**). MT, as a ‘suicide’ antioxidant, can directly scavenge radicals and radical products; additionally, MT indirectly scavenges radicals by stimulating protective antioxidant enzymes, suppressing pro-oxidant enzymes, and improving mitochondrial function, thereby reducing radical formation (Zhang and Zhang, 2014). On the other hand, polyphenols can act as an antioxidant (Zhang and Tsao, 2016); in grape berries, the content of total phenols, flavonoids, anthocyanins, and proanthocyanidins exhibited a significant correlation with antioxidant properties (Xu et al., 2010). The enhanced polyphenol content, including total phenols, anthocyanins flavonoids, proanthocyanidins and individual phenolic compounds, might contribute positively to antioxidant capacity in MT berries (**Tables 1**–**3**). However, to date, there are no reports directly linking MT levels and polyphenol content, although a possible association between them was suggested. For example, both MT and total polyphenol content were higher in wine produced from grapes treated with resistance inducers than in those obtained from untreated controls (Vitalini et al., 2011). In contrast, this study provided the first evidence that MT enhanced antioxidant capacity, at least partially, via promoting polyphenol accumulation.

The large changes in non-flavonoid, flavanol and anthocyanin compounds were also observed in the MT-treated berries (**Tables 2**, **3**). Epicatechin, syringic acid and gallic acid were the three highest levels of phenolic compounds in ‘Moldova’ grapes (**Tables 2**, **3**). Additionally, it was reported that the free radical scavenging activity of gallic acid, catechin and epicatechin are higher and they show high correlations with antioxidant activities compared to other phenolic compounds (Alvarez-Casas et al., 2016). Therefore, the significant increases in epicatechin and gallic acid might contribute positively to the enhancement of antioxidant capacity in MT berries. It should be noted that different cultivars possess different compositions of phenolic compounds; e.g., gallic acid and caftaric acid were the most abundant phenols in ‘Sauvignon blanc’ (Landrault et al., 2001), whereas ‘Chardonnay’ in the Galician region is characterized by its high levels of catechin and epicatechin (Li et al., 2009). Therefore, the different responses of polyphenols to MT might be diverse among grape cultivars. On the other hand, anthocyanins are described as main contributors to *in vivo* antioxidant capacity in grapes (Lingua et al., 2016a). Anthocyanins are glycosides of anthocyanidins, and six different anthocyanidins are found in nature; i.e., pelargonidin, cyanidin (Cy), delphinidin (Dp), peonidin, petunidin, and malvidin (Mv). In some hybrids of *V. labrusca* ×*vinifera* including ‘Moldova’ grapes, Mv-derivatives, such as Mv-3-(6-coumaryl)Glu-5-glu and Mv-3,5-Glu, are the most abundant components (**Table 3**; Liang et al., 2008); however, Cy-derivatives and Dp-derivatives were reported to be the most abundant anthocyanins in other cultivars of *V. labrusca* ×*vinifera* (Liang et al., 2008), indicating the large difference in anthocyanin composition in these hybrids. In contrast, cyanidin derivative (Cy-3-Glu) showed the lowest concentration in ‘Moldova’ grapes (**Table 3**), probably because this anthocyanin is the precursor of all others (Núnez et al., 2004). Malvidin possesses great antioxidant capacity with excellent free radical scavenging properties *in vitro* and in cells (Huang et al., 2016). Mv-3,5-Glu might be one of the primary contributors to the increased antioxidant activity in MT berries, based on its high concentration which accounted for more than 59.2% of the detected total anthocyanins (**Table 3**). It is noteworthy the accumulation of total anthocyanins was largely slowed down at 77 DAT (**Figure 1B**) and a decline in total anthocyanins and anthocyanin compounds was reported to occur at full maturation and/or during over-ripening (Ryan and Revilla, 2003). Therefore, it is suggested that MT accelerates berry ripening, likely lead to over-ripened berries, and hence altered anthocyanin metabolism compared to the CK berries.

Additionally, the antioxidant capacity is the result of synergistic or antagonistic effects from interactions between different polyphenol compositions among each other and with other components of the food matrix or organism; for example, it was also reported that Mv-3-Glu showed a synergistic antioxidant effect with catechin on free radical-initiated peroxidation of linoleic acid in micelles (Rossetto et al., 2002). Therefore, the enhancement of antioxidant capacity induced by MT is associated not only with the increased endogenous MT and polyphenolic constituents but also with their complicated reactions.

## Conclusion

This is the first report to elucidate that polyphenol metabolism is the most predominant biological process in response to MT treatment in grape berries in the light of RNA-Seq analysis. We also found that two melatonin treatments of veraison grape berries increased the contents of total anthocyanins, phenols, flavonoids, and proanthocyanidins. Meanwhile, the MT-induced increases in individual phenolic compounds were observed, including chlorogenic acid, gallic acid, epicatechin and Mv-3,5-Glu. In addition, MT treatment enhanced the antioxidant capacity of berries. Furthermore, ethylene was indicated to participate in the regulation of polyphenol metabolism and antioxidant capacity under MT treatment in grape berries.

## Author Contributions

YY and HZ conceived and designed the experiments. LX, QY, and FB carried out the experiments. LX and HS performed data analysis. YY and HZ wrote the paper.

## Conflict of Interest Statement

The authors declare that the research was conducted in the absence of any commercial or financial relationships that could be construed as a potential conflict of interest. The reviewer AS and handling Editor declared their shared affiliation, and the handling Editor states that the process nevertheless met the standards of a fair and objective review.

## Footnotes

1 http://genomes.cribi.unipd.it/grape/
