# Supplementary material for: Melatonin Enhances Phenolics Accumulation Partially via Ethylene Signaling and Resulted in High Antioxidant Capacity in Grape Berries
Source: Front Plant Sci. 2017 Aug 18;8:1426. doi: 10.3389/fpls.2017.01426 (PMC5563355; doi:10.3389/fpls.2017.01426)
Supplement: Supplementary file 1 [file Table_1.DOCX]

**Supplementary Table 1 | Primers used for real-time quantitative PCRs.**

| **Genes** | **Forward primers (5ʹ-3ʹ)** | **Reverse primers (5ʹ-3ʹ)** |
| --- | --- | --- |
| *VIT_200s2849g00010(PAL)* | AAGATCATCGACCCTCTTCTG | GAAAGCGTGCAGACAATCTC |
| *VIT_216s0039g01360(PAL)* | GAATGACATGTCCTAGGAGCTG | CAGACCAACAAATACTTCCTAAGG |
| *VIT_216s0039g01130(PAL)* | TGGATTGTCTCAGTGCTTGG | AGCGTGCTGACAATCTCAAG |
| *VIT_204s0023g02900(F5H)* | TCAAGATCATGGAGGGACTG | CAAATCTCCTGTTCAACATCAAC |
| *VIT_216s0100g01130(STS)* | CATCGGTGAAGGATTGGATTG | ACATTGAAGGGTCGAGCATTC |
| *VIT_216s0100g00780(STS)* | TACAGAGGAGGTGCTACGAAAG | CTTGCCTGACGTGACTGC |
| *VIT_216s0100g00810(STS)* | GAAAAACCATTCACGAGCCA | AGGTCTCTGAAAAAGTCCTCCAA |
| *VIT_216s0100g01150(STS)* | CAGGTGAAGGATTGGATTG | CAACATTAAAGGTACCATTCTCC |
| *VIT_216s0100g00990(STS)* | AGAATGGTCCCTTCAATGTCC | GTTTCATTTGAGCTCACCCAG |
| *VIT_210s0003g00480(ROMT)* | GTTACTCTGCAGCATGGTCAC | AGACAGACACAACCAAGTAACAAC |
| *VIT_209s0096g00680(HCT)* | ACCGTCTCAATTTGCTTAACG | GCTAGGAGGCATTTATGCTTG |
| *VIT_206s0009g02920(F3'5'H)* | TGAGATCAACATGGACGAAGC | TTGGTCTGAGCGGTTTGAG |
| *VIT_207s0005g03340(MYB4)* | GATTGGACAACAATTATCGGTG | CTGCAAGTCATTCTCAATCAACA |
| *VIT_202s0025g00360(ACS)* | ACCGTCTTTTCTCTCTCGCC | TCTTGGACAACAACTGCGGT |
| *VIT_215s0046g02220(ACS)* | TGTGATGTCCCCTCATTCCC | CGCATCTAACTGCCAACCAA |
| *VIT_211s0016g02380(ACO)* | CCTGTTCTCATCATCCTGGGTC | AGCCTTTGTTTCAGCCCTTT |
| *VvACTIN* | TCTCAACCCAAAGGCTAATC | GCATAGAGGGAAAGAACAGC |
