# Supplementary material for: Melatonin Enhances Phenolics Accumulation Partially via Ethylene Signaling and Resulted in High Antioxidant Capacity in Grape Berries
Source: Front Plant Sci. 2017 Aug 18;8:1426. doi: 10.3389/fpls.2017.01426 (PMC5563355; doi:10.3389/fpls.2017.01426)
Supplement: Supplementary file 3 [file Table_3.DOCX]

**Supplementary Table 3 | Statistical effects of year, MT treatment and their interaction on polyphenols and antioxidant capacity.**

| **Tissues** | **Parameters (unit)** | **2015** | | **2016** | | Year  (*p* value) | MT treatment  (*p* value) | Year × MT treatment (*p* value) |
| --- | --- | --- | --- | --- | --- | --- | --- | --- |
|  |  | **CK** | **MT** | **CK** | **MT** |  |  |  |
| Pulp and skin | Melatonin (ng.g^-1^ DW) | 1.33 | 1.87 | 1.45 | 1.92 | 0.334 | 0.001 | 0.627 |
| Pulp | TSS | 15.60 | 16.81 | 16.07 | 17.16 | 0.097 | 0.001 | 0.808 |
| Skin | Total anthocyanin (Relative unit) | 46.14 | 58.56 | 46.77 | 60.13 | 0.267 | 0.000 | 0.830 |
| Pulp | Titratable acid (mg. tartarate. ml^-1^ juice) | 5.99 | 4.98 | 5.61 | 5.13 | 0.662 | 0.021 | 0.346 |
| Skin | Total phenols (mg gallic acid.g^-1^ FW) | 3.65 | 4.91 | 2.94 | 4.15 | 0.016 | 0.001 | 0.926 |
| Skin | Total flavonoids (mg rutin.g^-1^ FW | 13.10 | 14.93 | 13.94 | 16.21 | 0.113 | 0.009 | 0.723 |
| Skin | Total proanthocyanins (mg vanillin.g^-1^ FW) | 80.32 | 96.95 | 75.32 | 89.17 | 0.039 | 0.000 | 0.548 |
| Skin | Gallic acid (mg.g^-1^ DW) | 1.44 | 2.65 | 1.63 | 2.65 | 0.442 | 0.000 | 0.442 |
| Skin | Coumaric acid (mg.g^-1^ DW) | 4.82 | 6.25 | 3.73 | 5.61 | 0.024 | 0.001 | 0.492 |
| Skin | Chlorogenic acid (mg.g^-1^ DW) | 0.70 | 1.24 | 0.74 | 1.46 | 0.144 | 0.000 | 0.289 |
| Skin | Trans-Resveratrol (mg.g-1 DW) | 1.15 | 2.28 | 1.64 | 2.65 | 0.021 | 0.000 | 0.684 |
| Skin | Mv-3,5-Glu | 15.20 | 27.95 | 13.41 | 24.16 | 0.050 | 0.000 | 0.430 |
| Skin | Mv-3-Glu | 1.83 | 2.25 | 2.12 | 2.74 | 0.016 | 0.004 | 0.466 |
| Skin | Pn-(6-Caff)Glu | 1.71 | 6.25 | 1.97 | 5.34 | 0.260 | 0.000 | 0.063 |
| Skin | DPPH(1/EC50) (mg gallic acid g^-1^ FW) | 0.18 | 0.24 | 0.23 | 0.33 | 0.011 | 0.000 | 0.210 |
| Skin | ABTS (mg Trolox g^-1^ FW) | 64.36 | 72.31 | 65.81 | 77.32 | 0.289 | 0.009 | 0.552 |
| Skin | FRAP (mg Trolox g^-1^ FW) | 83.06 | 102.85 | 86.26 | 102.96 | 0.582 | 0.000 | 0.675 |

*Values are determined using the berries at 63 DAT and reported as the means of the three replicates. DW, dry weight.*
